# Active Surfaces Formed in Liquid Crystal Polymer Networks

**DOI:** 10.1021/acsami.1c21024

**Published:** 2022-02-10

**Authors:** Mert O. Astam, Yuanyuan Zhan, Thierry K. Slot, Danqing Liu

**Affiliations:** † Laboratory of Stimuli-Responsive Functional Materials and Devices (SFD), Department of Chemical Engineering and Chemistry, Eindhoven University of Technology, Groene Loper 3, Eindhoven AE 5612, The Netherlands; ‡ Institute for Complex Molecular Systems (ICMS), Eindhoven University of Technology, Groene Loper 3, Eindhoven AE 5612, The Netherlands; § SCNU-TUE Joint Lab of Device Integrated Responsive Materials (DIRM), National Center for International Research on Green Optoelectronics, South China Normal University, Guangzhou 510006, P. R. China

**Keywords:** soft robotic functions, dynamic surface topographies, liquid secretion, liquid crystal polymer networks, free volume

## Abstract

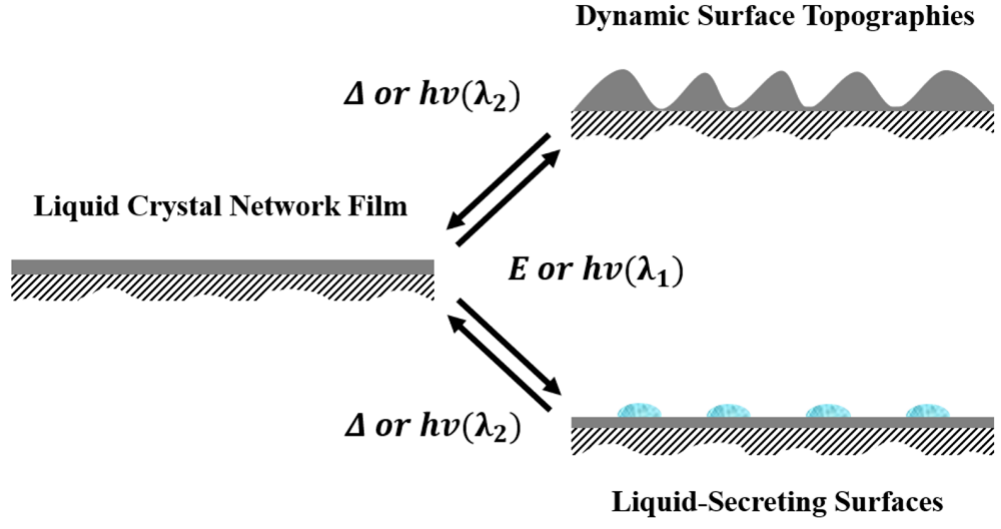

There is an increasing interest in animating materials to develop dynamic surfaces. These dynamic surfaces can be utilized for advanced applications, including switchable wetting, friction, and lubrication. Dynamic surfaces can also improve existing technologies, for example, by integrating self-cleaning surfaces on solar cells. In this Spotlight on Applications, we describe our most recent advances in liquid crystal polymer network (LCN) dynamic surfaces, focusing on substrate-based topographies and dynamic porous networks. We discuss our latest insights in the mechanisms of deformation with the “free volume” principle. We illustrate the scope of LCN technology through various examples of photo-/electropatterning, free-volume channeling, oscillating/programmable network distortion, and porous LCNs. Finally, we close by discussing prominent applications of LCNs and their outlook.

## Introduction

Liquid crystal networks (LCNs) are polymer networks with tunable optical, mechanical, and electrical properties.^1−8^ LCNs have found applications in extensively commercialized liquid crystal display technology.^9−12^ But, as this field reaches maturity, the use of LCNs as stimuli-responsive materials for soft actuators and sensors has moved into the spotlight. Its emerging functions include tribology (friction and wear), microfluidics, and switchable (super)hydrophobicity.^13−18^ With these new functionalities, LCNs can be applied to improve existing technologies, such as solar cells (in coupling of sun light, self-cleaning), architecture (self-cleaning, light/heat regulating), and smart windows (efficiency, climate control).^13,19−21^ This technology also opens up new avenues to robotic handling of chemicals, drug transfer, and surface lubrication.^22−24^

In this Spotlight on Applications, we focus on our recent advances within the field of stimuli-responsive liquid crystal surfaces. We evaluate active dynamic surfaces in two categories: densely cross-linked dynamic surface topographies and porous liquid-secreting surfaces. We first introduce the patterned-alignment-based geometric deformation principles of LCNs and their role in the creation of both dynamic surface topographies and liquid-secreting surfaces. Second, we discuss “free volume”-based phenomena and deformation principles. Finally, we close this review with a future-oriented overview of the applications of both densely linked and porous LCN materials.

## Anisotropic Deformation of Dynamic Surface Topographies

**Figure 1:**
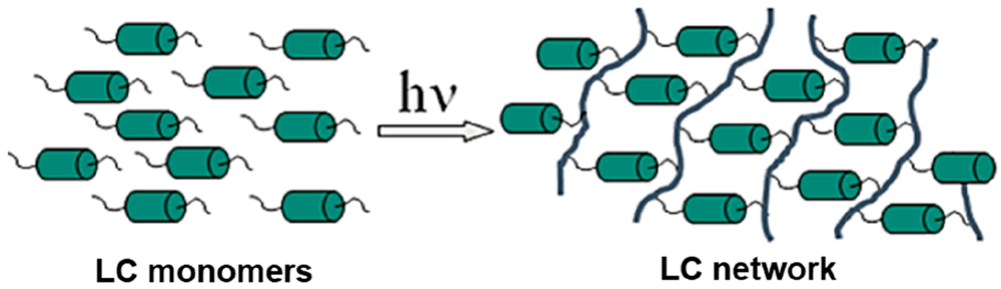
Schematic of photopolymerization arresting the nematic state molecular configurations. Reproduced with permission from ref (29). Copyright 2014 American Chemical Society.

Liquid crystal networks (LCNs) are commonly synthesized by photopolymerizing (Figure 1) reactive mesogens. Examples of monoacrylate and diacrylate reactive mesogens are given in Figure S1a–d.^25^ Mixtures of reactive mesogens are often formulated to control monomer properties, for instance, nematic phase transition temperature, allowing for room-temperature processing. This can also be used to control mechanical properties of the resulting polymer, for example, elastic modulus and glass transition temperature. In the monomeric state, prior to the polymerization of the network, various molecular configurations can be established by methods developed in the liquid crystal display industry.^26,27^Figure S2 shows a few molecular alignments, namely, twisted nematic, splay-bend, tilted uniaxial, helicoidal chiral-nematic (cholesteric), or homeotropic.^28,29^

Upon decreasing the order of the established molecular alignment, the polymer network contracts along the general molecular orientation axis (director) and expands in the perpendicular direction.^30−32,48^ Thereby, actuation can be achieved. This can be done using an external stimulus, such as temperature, electric field, or light.^33−46^ In the case of electrical-driven systems, reactive mesogens with large dipole moments are used, an example of which is shown in Figure S1a. As for light-triggered systems, LCNs are doped with light-responsive additives, such as azobenzene. Azobenzene molecules isomerize to their bent cis states upon UV illumination, which reduces the molecular order of LCNs (Figure S1e). The resulting actuation can be relaxed thermally or, using blue light, convert cis azobenzene molecules back to their initial trans states, where they are compliant with the LCN. On the basis of this principle, many stimuli-responsive 3D deformation modes, or standalone elements, have been demonstrated using LCNs with various alignment set-ups.^43,47^

For our research, we mainly focus on coating configurations and the corresponding surface deformations, shifting from 3D actuation to 2D dynamics. In coatings, in-plane deformations are restricted by the confinement of the thin film on a rigid substrate. To create these dynamic surfaces, we designed complex molecular architectures by spatially combining two or more alignments within the liquid crystal mesophase, enabling control of the actuation direction and amplitude. An example of this is a film with alternating strips of chiral nematic and isotropic phases. The regions of chiral nematic order have, on average, planar alignment, whereas the molecules in the isotropic regions have no alignment. Therefore, upon actuation, the regions with chiral nematic order expand along the direction of the coating thickness, whereas the isotropic areas exhibit negligible deformation (Figure S3a, b). This produces a deformation strain of 5–10% perpendicular to the substrate plane. The strain is reversible and the initial surface can be retained by removing the stimuli.^50^

**Figure 2:**
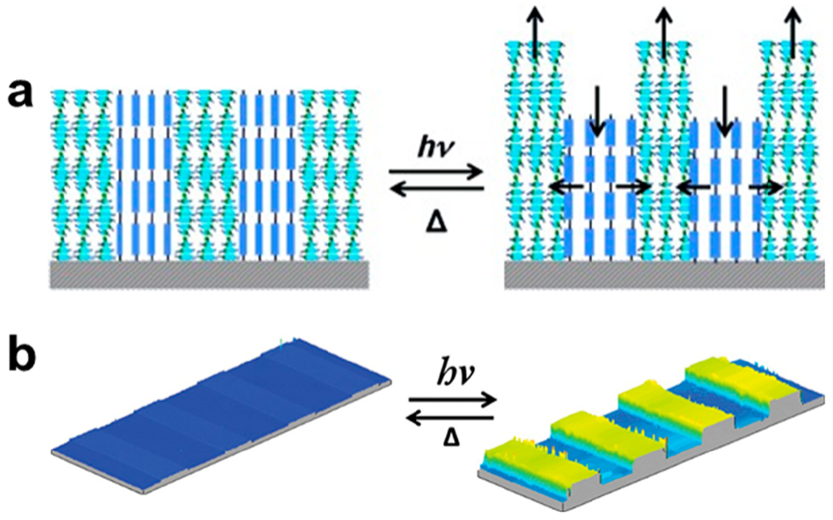
(a) Schematic of photoinduced actuation of strips of alternating chiral nematic order and homeotropic alignments. Reproduced with permission from ref (51). Copyright 2011 John Wiley and Sons. (b) Interference microscope images of resulting topography. Reproduced with permission from ref (51). Copyright 2011 John Wiley and Sons.

To further enhance the deformation amplitude, the nonaligned, isotropic areas were replaced by regions with homeotropic alignment. The objective of this modification was to create a synergistic double action, as the homeotropically aligned regions were expected to contract in the opposing direction to the planarly aligned nematic regions. This effectively pinched the nematic regions to create peaks, resulting in a deformation strain of almost 20% (Figure 2a, b).^51^

**Figure 3:**
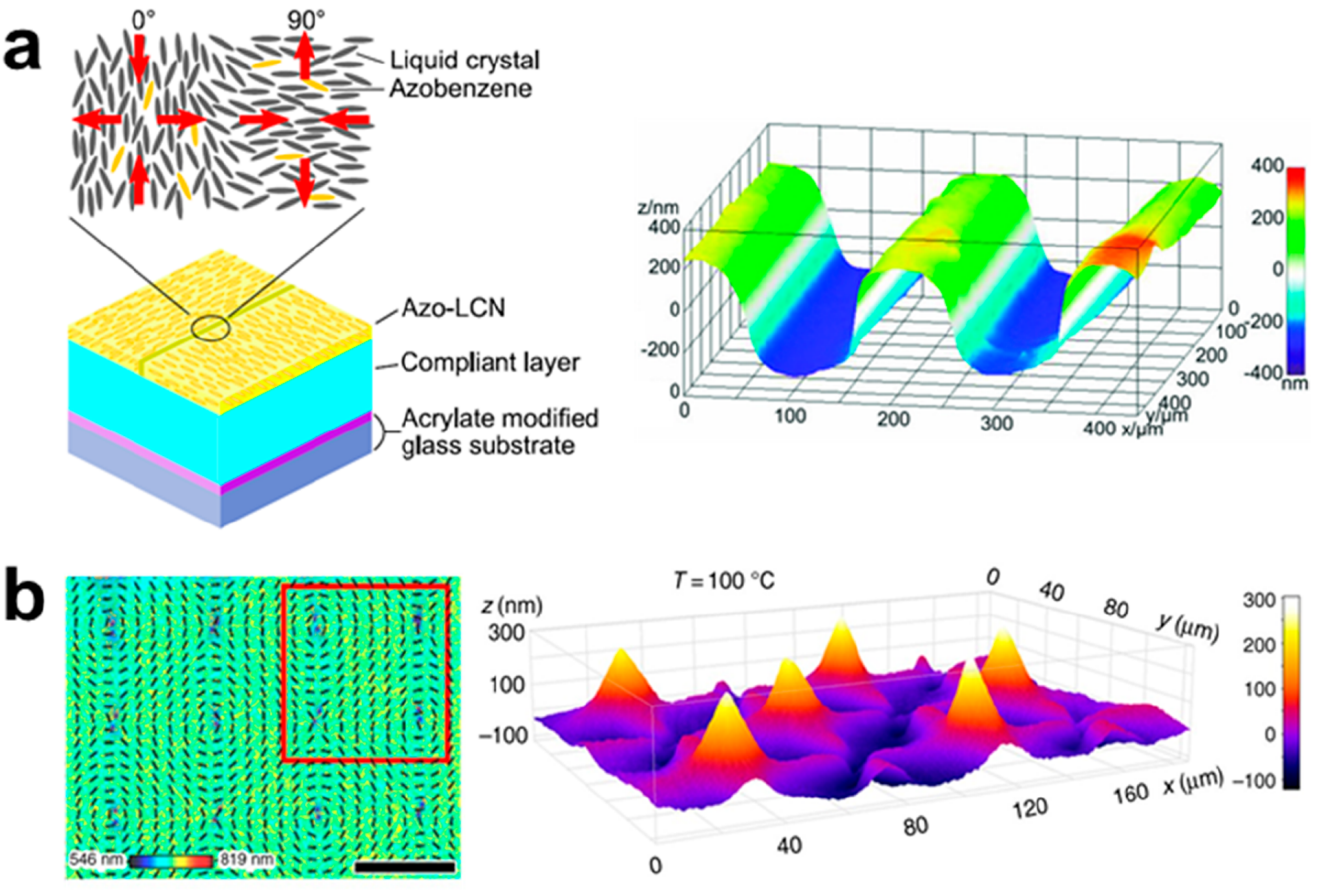
(a) 3D visualization of domain configurations and topography in compliant substrates. Reproduced with permission from ref (52). Copyright 2018 John Wiley and Sons. (b) Digital schematic image of LCN coating surface with director orientation with circular defects with corresponding digital holographic microscopy (DHM) surface image. Reproduced with permission from ref (53). Copyright 2018 Nature Research.

Beyond patterning alignment, replacing the rigid substrate with a compliant substrate can also enhance deformation by increasing the freedom for vertical deformation (Figure 3a).^52^ Further expanding the concept of patterned alignments to locational alignments, photopatterning was used to induce surface defects in 3D topographies. Specifically, 2D radial defects were shown to lead to surface depressions, whereas 2D circular defects were shown to lead to surface elevations (Figure 3b). This allows for the integration of different actuation configurations across the same LCN film.^53^

**Figure 4:**
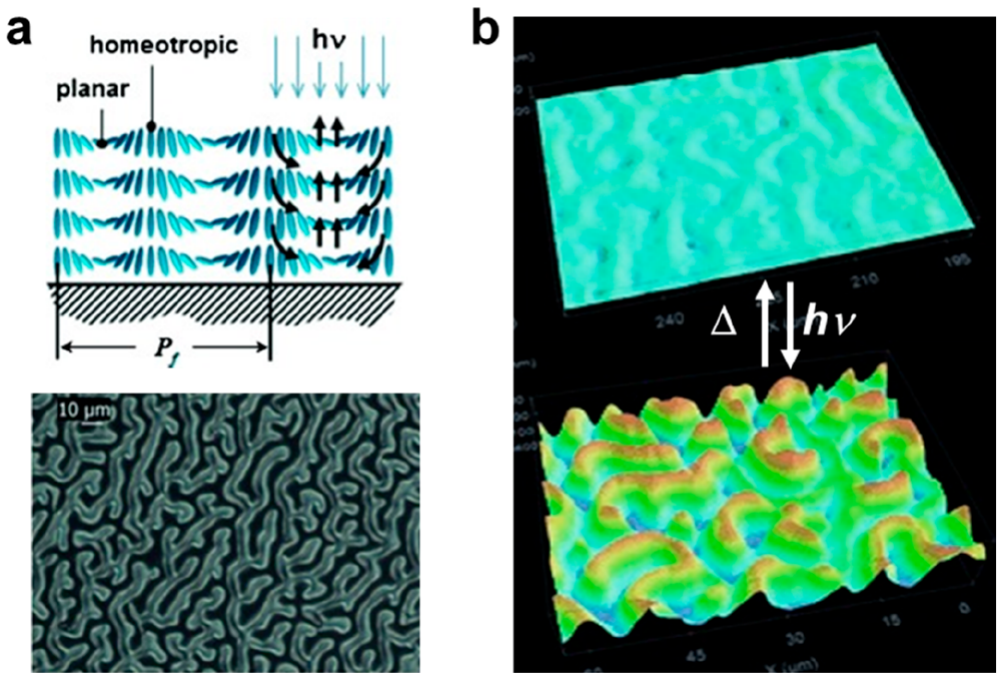
(a) Schematic of photoinduced actuation of LC helices. Reproduced with permission from ref (14). Copyright 2014 John Wiley and Sons. (b) Polarized optical microscopy images of resulting “fingerprint” topography. Reproduced with permission from ref (14). Copyright 2014 John Wiley and Sons.

Locational alignment can also be achieved by using chiral reactive mesogen molecules to obtain a helical LC phase. For instance, in “fingerprint” textures, where the helix axis is parallel to the substrate, the molecules make a full rotation of 360° along this axis. This is shown by the corresponding polarized optical microscopy (POM) image (Figure 4a, b), where regions with planar alignment are represented by brighter pixels and areas with homeotropic alignment are displayed by darker pixels. Initially, this surface exhibits close-to-flat topography. Specifically, the free energy differences between homeotropically aligned and planarly aligned molecules induce the Marangoni effect in the initial liquid, monomeric LC mixture, resulting in small reliefs that are then arrested by the subsequent photopolymierzation.^54^ Upon actuation, the planarly aligned regions form protrusions, whereas homeotropically aligned regions form indentations. As a result, a 3D “fingerprint” topography emerges, with peaks and valleys forming adjacent channels, akin to human fingerprints.^14^

**Figure 5:**
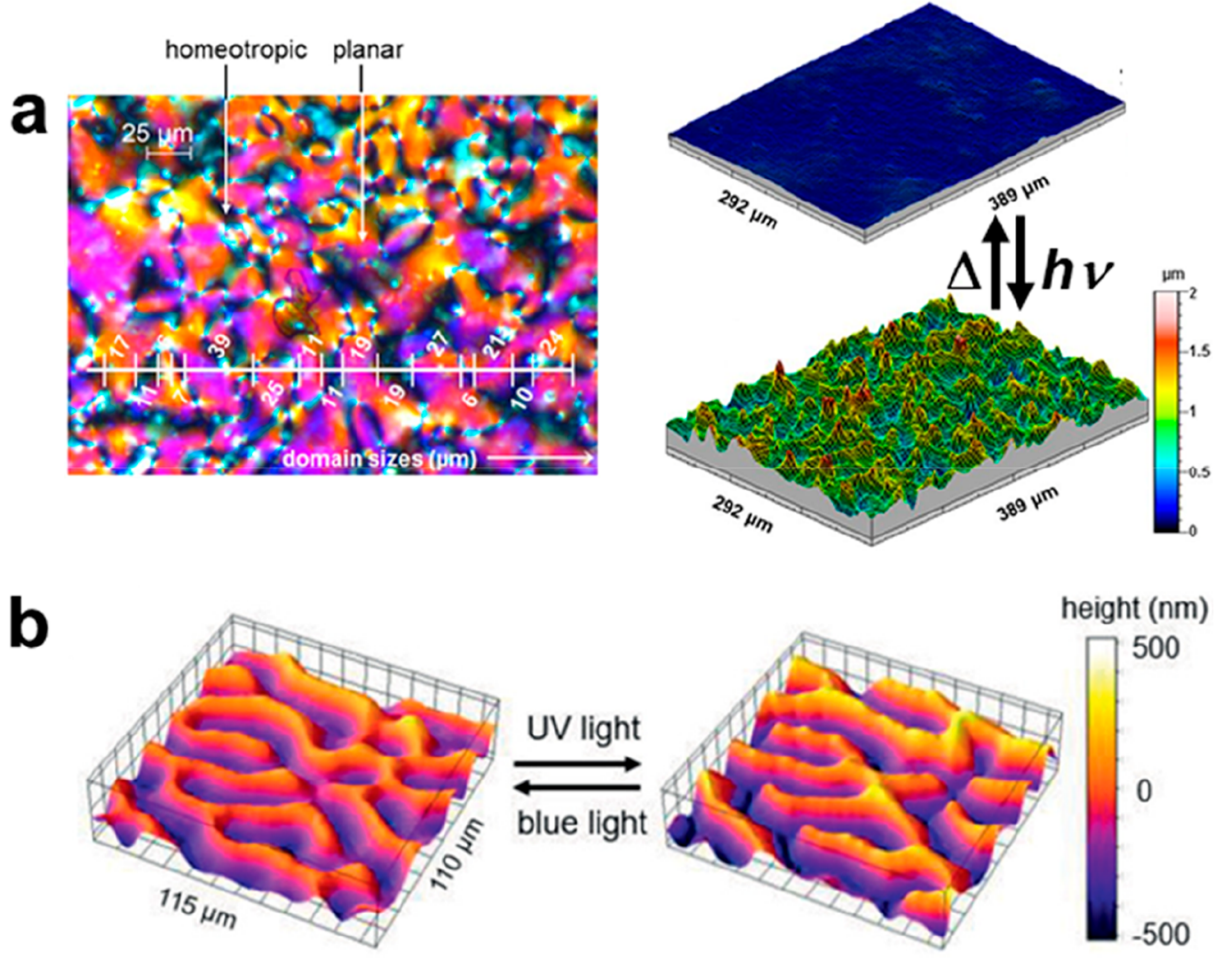
(a) POM image of polydomain configurations and its corresponding topography upon actuation. Reproduced with permission from ref (15). Copyright 2015 United States National Academy of Sciences. (b) DHM image representation of light-responsive topographical inversion. Reproduced with permission from ref (16). Copyright 2021 John Wiley and Sons.

In the dynamic LCN films discussed so far, complex and expensive lithography or photopatterning is necessary to produce predesigned alignments. This can make adoption of the technology for applications difficult or too expensive.^52^ Therefore, we developed polydomain systems that involve simpler one-step coating methods, requiring no additional pretreatment on the substrate (Figure 5a). Actuating polydomain systems causes homeotropically aligned regions to form valleys and planar-aligned regions to form peaks, whereas domain borders with mixed alignment display variable deformation. This results in a rough surface with a spiky topography.^15,18^

Hitherto, coatings that transition between a flat state and a predesigned corrugated state have been discussed. This functionality was further explored by inverting the topography of a LCN film with an initially corrugated surface. Fundamentally, the initial peaks become valleys and vice versa (Figure 5b). This was performed in a dichroic-dye-doped “fingerprint” LCN film, where the helical LC phase induced by the chiral dopants result in alternating planar and homeotropic regions. Crucially, the integrated dichroic dyes absorb light predominantly in planar areas, which results in faster polymerization in the homeotropic regions. This transports material from the planarly to homeotropically aligned regions during photopolymerization, in a so-called polymerization-induced diffusion process. Thereby, homeotropically aligned regions accumulates more material and form peaks, whereas planarly aligned regions form valleys. Upon a reduction in the order parameter, the homeotropically aligned regions contract vertically and planarly aligned regions contract horizontally with respect to the plane of the substrate. As a result, the initial homeotropically aligned peaks sink back toward the substrate, whereas the planarly aligned valleys contract and “bunch-up”. This allows for a film that can switch between two functional states, the applications of which are elaborated upon in the applications section.^16^

The tailorable, highly stimuli-responsive actuating properties of LCs are not only valuable for dynamic topographies in cross-linked LCNs but also in liquid-secreting porous LCNs or LC gels. These spongelike materials are based on higher-order smectic LCs with homeotropic alignment. Generally, they are prepared by mixing nonreactive LC liquid with reactive mesogens, followed by photopolymerization. The mixture phase-separates as the network forms, creating (sub)micrometer-sized pores filled with nonreactive LC liquid. The sponge can be considered as a gel with an adjustable liquid capacity.

**Figure 6:**
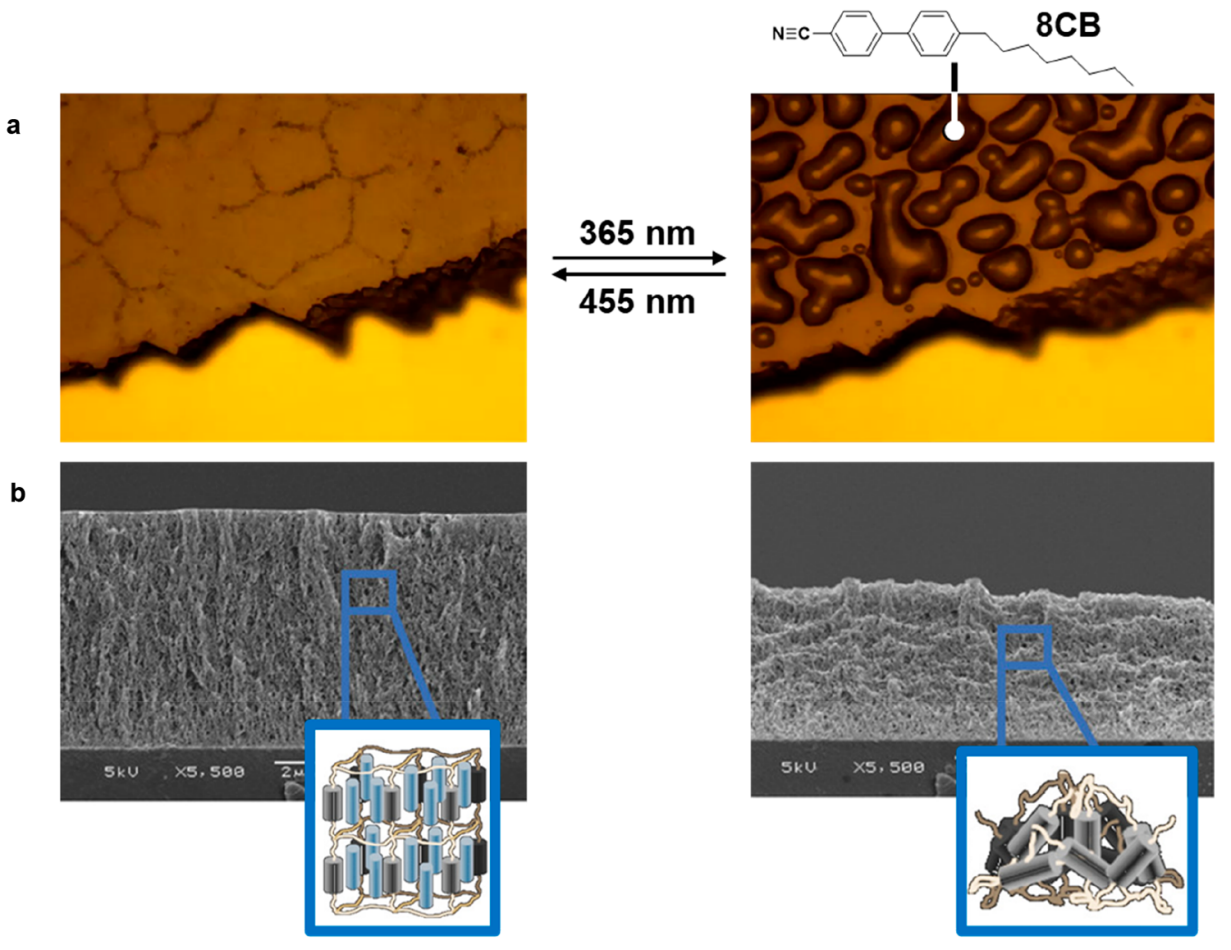
(a) Optical microscopy images of 8CB secretion and absorption in a porous LCN film upon UV illumination and blue light illumination, respectively. Reproduced with permission from ref (22). Copyright 2018 John Wiley and Sons. (b) POM pinpointing birefringent liquid secretion in an LCN photosponge film and corresponding SEM images of porous structure collapse with removal of liquid. Reproduced with permission from ref (23). Copyright 2020 American Chemical Society.

The sponge can be triggered using light, heat, or electricity. In the case of light-responsiveness, approximately 5 wt % azobenzene was added to the porous LCN to create a photosponge. Upon UV illumination, the molecular order parameter decreases and leads to contraction in the LCN film. This results in a reduction of the thickness dimension in the LCN and compresses the encapsulated liquid, squeezing it out of the bulk of the coating. Like a sponge, the secreted liquid can be readsorbed upon illumination with blue light. (Figure 6a, b).^22^

The discussed light-responsive sponge secretes liquid uniformly across the entire film. Some applications, such as very fine microfluidics, may require localized liquid secretion at predetermined points. This can be achieved with patterned LC molecular alignment, such as alternating homeotropically aligned smectic and nonaligned isotropic regions. A two-step photopolymerization process is needed: first, the smectic homeotropic regions with homeotropic alignment are captured by polymerization through a photomask, then the isotropic regions are established by heating the film above the smectic-to-isotropic transition temperature and photopolymerizing again to also arrest the lack of order in the isotropic regions. Upon actuation, liquid secretion is restricted to only smectic areas with homeotropic alignment (Figure S4) as these regions can experience changes in order parameter and thus actuate, unlike the isotropic regions which have no alignment.^23^

This sponge can also be electrically responsive (E-sponge) because of the large dipole moment of the 8CB porogen. To induce liquid displacement, we used an in-plane switching electric field underneath the film. When the E-sponge is activated, the retained polar 8CB porogen diffuses to the regions where the largest electric field strength appears to align its dipoles along the electric field lines. Then, once the displaced liquid builds up enough pressure, it is released to the surface. The on and off switching of the electric field is programmable, allowing for on-demand liquid secretion and reabsorption (Figure S5).^24^

## From Anisotropic Deformation to Free Volume

**Figure 7:**
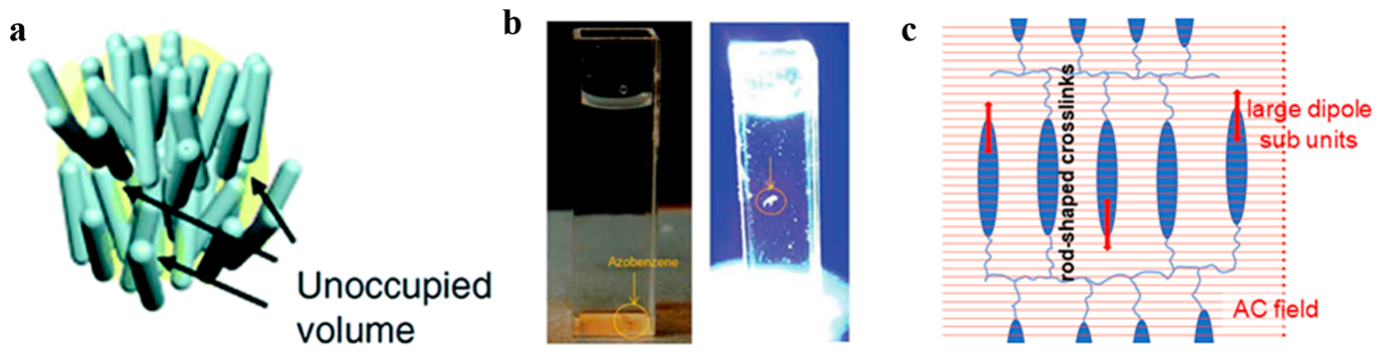
Schematic representations of free volume mechanisms with data and footage of the concept in action. (a) Illustration of “free volume” generated within an LCN network upon actuation. Reproduced with permission from ref (63). Copyright 2019 John Wiley and Sons. (b) Demonstration of “free volume”-induced density decrease with the ascent of an initially sinking immersed film upon irradiation. Reproduced with permission from ref (64). Copyright 2014 American Chemical Society. (c) Schematic representation of electrical-responsive LCNs based on the actuation of high-dipole molecules under an alternating-current electrical field. Reproduced with permission from ref (65). Copyright 2017 Nature Research.

The relaxation rate mismatch between LCN and its azobenzene dopant challenged the classic order-parameter-based deformation model. Fundamentally, the classic deformation model dictates that the deformation in LCNs is directly coupled to its current order parameter, which means the deformation would respond immediately to any change in order parameter and vice versa.^55−57^ Thus, using this model would lead to the prediction that the relaxation time of a LCN should be coupled to that of azobenzene. Specifically, as azobenzene isomerization changes the overall order parameter of the system, the LCN should actuate and remain in the actuated state for as long as its azobenzene moieties retain their new cis isomerization state. Yet, the azobenzene-doped LCN topography was observed to relax within 10 s of the stimuli being removed, whereas the relaxation of azobenzene molecules from cis to trans states required hours or days in the dark. Hence, the classical model makes an incorrect prediction in this case. Therefore, we proposed a new mechanism based on “free volume” generation (Figure 7a), where energetically unfavorable free volume is created by decreasing the order parameter and disappears spontaneously with removal of stimuli. Thus, “free volume” is a dynamic property related to the “excluded volume” concept, yet the motion of the polymer chains originate from, and is influenced by, their interaction at the alignment level rather than random vibrations in solution.^58^ To verify this, an experiment measuring the in situ density of an azobenzene-doped LCN film was designed. As was demonstrated by the LCN film floating in a liquid medium after UV illumination (Figure 7b), the film density was shown to decrease with actuation.^48^ This shows that the volume of the LCN film increases with decreasing order parameter, marking free volume as an important factor in LCN actuation.^49^ On the basis of this discovery, a vertically swimming LC seas-slug was developed by triggering density decrease upon actuation by UV illumination. The author of this paper reports a density decrease of around 8%, which agrees with our proposed mechanism.^59^

Building upon our discovery, dynamic free volume generation was found to be enhanced by. adjusting the trans–cis/cis–trans isomerization dynamics of azobenzene. Our experiment demonstrated that this can be achieved by illuminating an azobenzene-doped LCN film with a combination of low-intensity blue light and higher-intensity UV light, which led to a 4-fold increase in deformation strain compared to using UV light alone. Thus, considering the free volume concept, this phenomenon suggests that the free volume generation originates from the continuous oscillation of the azobenzene from its trans-state to its cis-state, and vice versa, by exposure to a combination of blue light and UV light, respectively. Doping an LCN film with fluorescent dye further confirmed this concept and enabled the tuning of the light intensities to achieve maximum azobenzene oscillation, as the fluorescent dye absorbed a small amount of UV light while emitting a low intensity of blue light in response. We hypothesize that the azobenzene oscillation caused by the combination of blue and UV light matches the eigen frequency of the polymer network, generating maximum free volume.^48,52,60^

Inspired by this new mechanism, we hypothesize that electrical actuation can influence free volume in a similar way. Here, polar molecules interact with an alternating electric field and start oscillating, influencing the molecular order and free volume (Figure 7c). As an added benefit, electrical actuation offers a high level of control over LCN deformation, because the electrical field amplitude and frequency can be easily tuned. Using this approach, we reached approximately 10% protrusion formation.

Typically, we can generate this degree of protrusion formation in both light and electrically driven systems. Yet, this number is larger than what is normally expected from a glassy polymer network. To understand this, we used in situ dynamic mechanical thermal analysis to monitor the modulus changes under UV illumination.^61^ To complement this, we also used time-resolved laser speckle imaging to observe time-specific changes in the dynamics of the polymer, such as in surface expansion and surface motion.^62^ We concluded from this measurement that the polymer network is plasticized during actuation, which shifts the glass transition temperature to a lower value. This process is reversible as the material then returns to its glassy state when the trigger is switched off. Analogous to the action of azobenzene oscillation, increasing the frequency enhances actuation, because more free volume is generated because of the increased molecular oscillations.

## Applications

In the past decades, we have put our theoretical knowledge into action to study a range of engineering aspects, including patterning techniques/actuation control, free-volume channeling, oscillating/programmable surface topographies by network distortion, and porous LCNs. We demonstrated that the stimuli-responsive, dynamic, and controllable tribology of 2D LCN films are useful in both dynamic and static soft robotics. Switchable properties based on chemical loading on selected topographies allow for deployment in both aqueous and dry environments.^66−68^ Moreover, the liquid-secreting LCNs can find biocompatible applications due to their low cytotoxicity.^69^ Many functions overlap in application, such as lubricating liquid-secreting LCN films and “fingerprint” films in the case of controllable friction.

**Figure 8:**
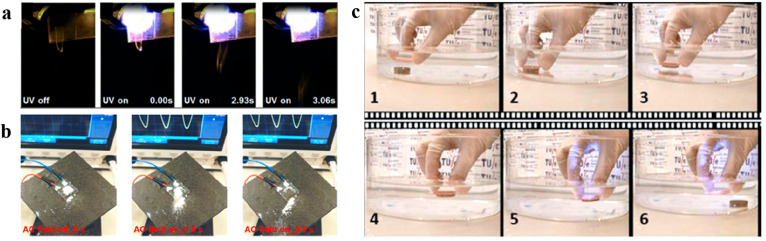
Snapshots and images of LCN applications based on controllable tribology. (a) Time-labeled footage of UV-triggered, gravity-assisted material release on a light-responsive LCN strip with switchable “fingerprint” topography. Reproduced with permission from ref (14). Copyright 2014 John Wiley and Sons. (b) Time-labeled footage of electrically triggered, gravity-assisted release of sand on an electrically responsive LCN strip with oscillating surface topography. Reproduced with permission from ref (13). Copyright 2018 John Wiley and Sons. (c) Snapshots of LCN film in action: a copper pellet is adhered on an LCN film and transported, and then the switchable “fingerprint” topography is activated by UV illumination to decrease friction for release. Reproduced with permission from ref (16). Copyright 2021 John Wiley and Sons.

In terms of soft robotics, controlled tribology takes an important role in robotic handling. Here, a number of developed approaches will be discussed. The first concept is based on modulated surface friction. The formation of LCN surface topographies with reduced contact area can decrease friction force between two surfaces for an easy release (Figure 8a). This technology can be applied to handle fragile objects.^15^ Soft grippers are typically applied for this, yet the compliant nature of the soft grippers can cause objects to adhere to the gripper and prevent release at the desired moment.^70^ LCN films can also be designed to oscillate its surface upon actuation, allowing for material to be mechanically repelled. This technique can also be used to create gravity-assisted, self-cleaning surfaces (Figure 8b).^13,14,65^ Another method to achieve controlled adhesion and release is by inverting the surface topography. For instance, when the peaks of only one topography are coated with adhesives, an adhesive and nonadhesive mode is created (Figure 8c).^16^ The coating applied on the switchable topographies can be selected to suit the desired operation environment.

Adhesion control can also be achieved using liquid for capillary bridge formation, which requires porous LCNs. Upon actuation by a stimuli such as heat or light, liquid is secreted and interacts with an opposing surface. It can induce adhesion by capillary bridging, or decrease friction if the secreted liquid acts as a microgap-filling lubricant (Figure S6a, b).^22^ For finer friction control, we can predesign locations for liquid secretion using preprogrammed patterned alignment.^23^

**Figure 9:**
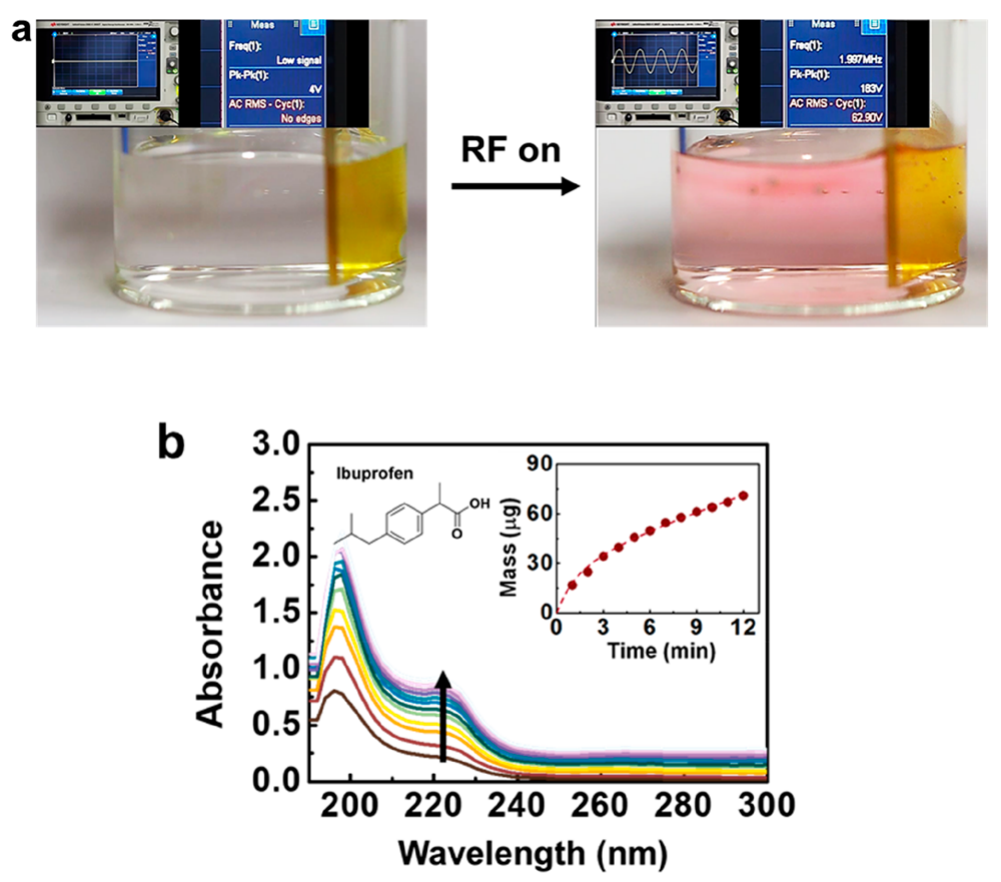
(a) Images of acid base reaction occurring with the secretion of pH-sensitive dye from an e-sponge upon activation by a radiofrequency electric field, as displayed in the oscilloscope images. Reproduced with permission from ref (24). Copyright 2020 Elsevier. (b) Graph presenting the release of ibuprofen from an activated LCN e-sponge over time. Reproduced with permission from ref (24). Copyright 2020 Elsevier.

Liquid-secreting LCN systems are stimuli-responsive chemical sponges, which can be applied in artificial skin, chemical sensors, self-regulating drug delivery, reagent release for chemical reactions and material transport, such as in heavy metal ion removal.^24,71,72^ Chemical reagent release has been demonstrated by the secretion of a pH-sensitive dye upon UV illumination of a photosponge (artificial skin). In this case, the reagent is released into an acid environment and undergoes a protonation-based color reaction at the acid/coating interface (Figure 9a). This function can also be tailored for the medical industry by replacing the pH-sensitive dye with a medical substance, such as ibuprofen. The secretion of the drug can be monitored by tracking its UV–vis absorption (Figure 9b).^24^ Nonorganic material transport is also possible, demonstrated by the diffusion of heavy metal ions from an LCN sponge into an aqueous environment.^72^

## Conclusion

LCNs can respond to many triggers such as temperature, electric fields, light, and even moisture, which grants them a lot of flexibility for real-world applications. This includes smart applications in self-cleaning surfaces, selectable and switchable surface properties, chemical and mechanical handling, sensors, and soft robotics. This is apparent even in this Spotlight on Applications, in which we focus mainly on our own work. Yet, there are many crucial contributions from worldwide prominent groups working in the field.^73−79^ Currently, the field focuses on advancing the theoretical knowledge of actuation, which is needed for creating fully functional devices in the future. We expect LCNs to become a staple technology in the design of such devices, especially in soft robotics as the field moves toward faster and finer actuating, corrosion-resistant, and biocompatible LCNs.

Invigorating discoveries in the theory of LCNs, such as the “free volume” theory, unlock new functions for LCNs, which can then be applied in the design of new devices. For instance, the concept of “free volume” was utilized to conceive LC aquatic robots. This demonstrates that LCN functionalization is still in its early stages, and has much greater potential to be released through further research both practical and theoretical.

However, for LCN devices to become useful in the real world, they must be upscaled to fit the scale of real-world applications. For example, the product of artificial skins need to have the LCN film upscaled from the mm range to the cm range. Moreover, the scale of not only the devices but also of LC manufacturing must be increased. For instance, to coat all of the world’s solar panels with self-cleaning LCN material would require an amount of reactive mesogens beyond the amount currently manufacturable. Nevertheless, the interest in producing LCN devices will increase with the growing expertise in its functionalization. Moreover, there are already companies with existing LC capacity, such as Philips and Merck for devices and chemicals production, respectively. Therefore, the infrastructure for upscaled manufacturing can be expected to be laid by such companies to take the LCN field to the next level of maturity.

## Supporting Information Available

Examples of mono- and diacrylates typically utilized to form LCNs; examples of molecular alignment; microscope, white light interferometer, and DHM images; footage of light-controlled adhesion and release and graph of corresponding film friction (PDF)

## Abbreviations

LC: liquid crystal
LCD: liquid crystal display
LCN: liquid crystal networks
DHM: digital holographic microscopy
POM: polarized optical microscopy.
