# Supplementary material for: Active Surfaces Formed in Liquid Crystal Polymer Networks
Source: ACS Appl Mater Interfaces. 2022 Feb 10;14(20):22697–705. doi: 10.1021/acsami.1c21024 (PMC9136844; doi:10.1021/acsami.1c21024)
Supplement: Supplementary file 1 — am1c21024_si_001.pdf [file am1c21024_si_001.pdf]

Supporting Information

Active surfaces formed in liquid crystal polymer networks

Mert O. Astam[1,2], Yuanyuan Zhan[1,2], Thierry K. Slot[1,2], Danqing Liu[1,2,3*]*

[1]Laboratory of Stimuli-Responsive Functional Materials and Devices (SFD), Department of Chemical Engineering and Chemistry, Eindhoven University of Technology, Groene Loper 3, 5612 AE Eindhoven, The Netherlands.

[2]Institute for Complex Molecular Systems (ICMS), Eindhoven University of Technology, Groene Loper 3, 5612 AE Eindhoven, The Netherlands

[3]SCNU-TUE Joint Lab of Device Integrated Responsive Materials (DIRM), National Center for International Research on Green Optoelectronics, South China Normal University, Guangzhou 510006, P. R. China.

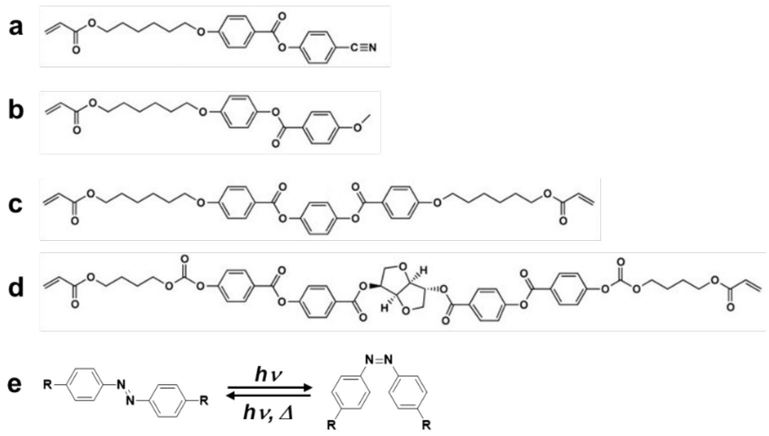

Figure S1. (a)-(c) Examples of mono- and di- acrylates typically utilized to form LCNs. (d) Example of a chiral di-acrylate typically utilized to form chiral nematic LCNs. (e) Illustration of the azobenzene molecule and its light-controlled *cis-trans* isomerization.

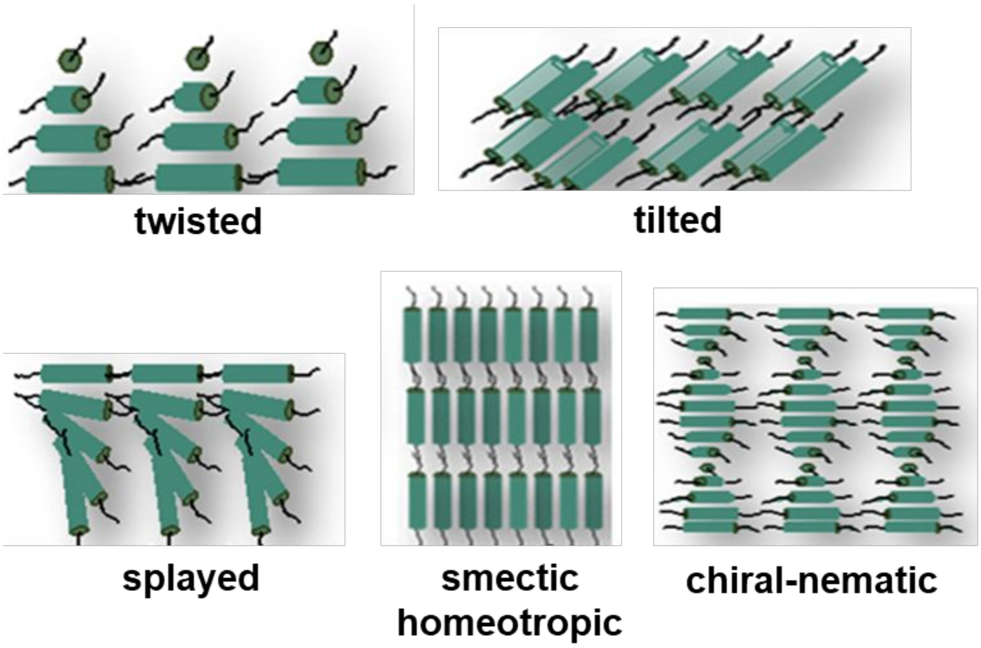

Figure S2. Examples of molecular alignment. Reproduced with permission from ref 29. Copyright 2014 American Chemical Society.

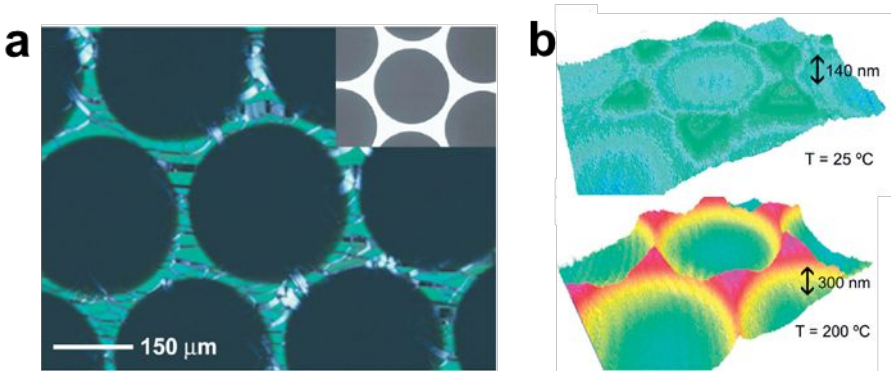

Figure S3. (a) Inserted microscope image of the photomask used to create circular isotropic regions on a cholesteric matrix. (b) White light interferometer images of resultant topography with thermal actuation. Reproduced with permission from ref 50. Copyright 2006 John Wiley and Sons.

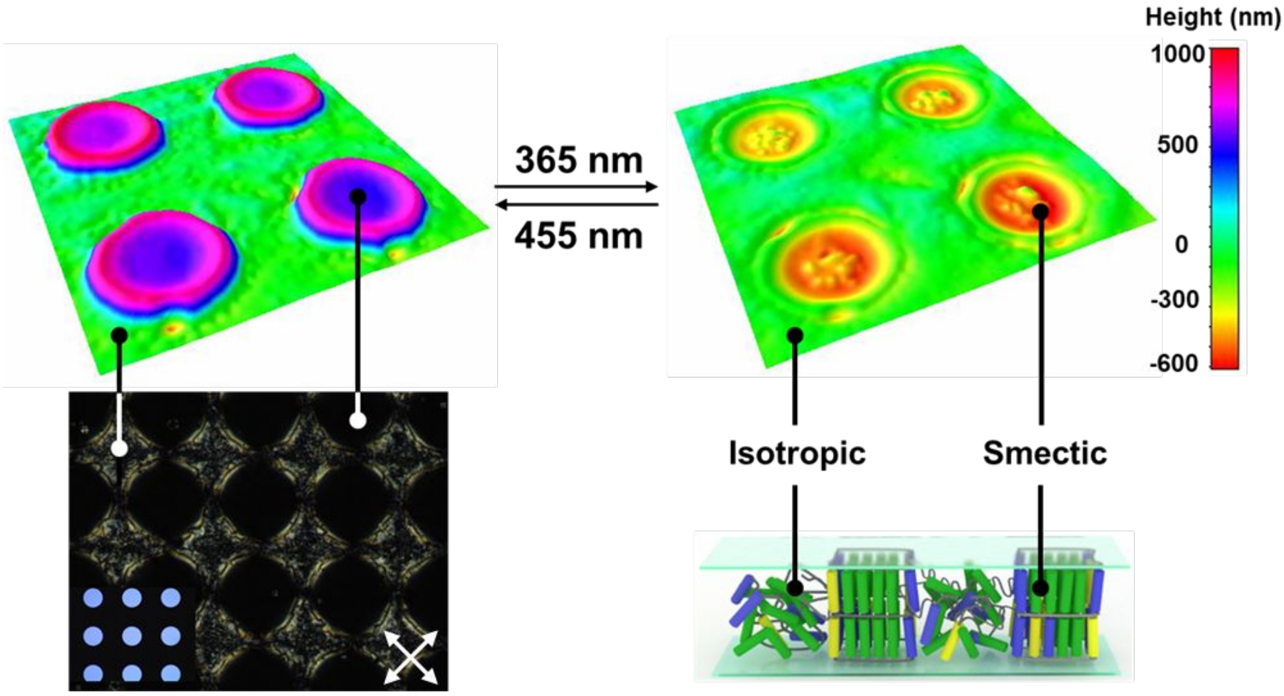

Figure S4. DHM images of photo-induced, homeotropic-alignment-localized liquid secretion in a porous LCN. Reproduced with permission from ref 23. Copyright 2020 American Chemical Society.

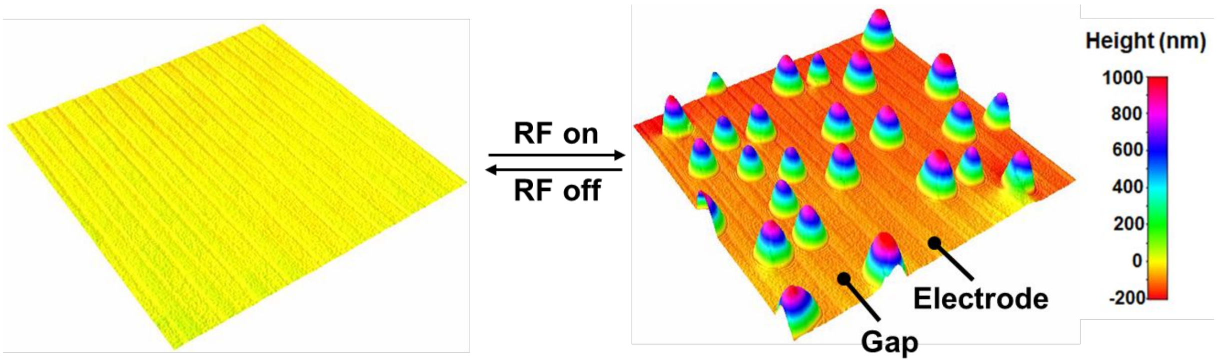

Figure S5. DHM images showing liquid secretion and absorption in a porous LCN e-sponge with the switching on and off of a radiofrequency electric field respectively. Reproduced with permission from ref 24. Copyright 2020 Elsevier.

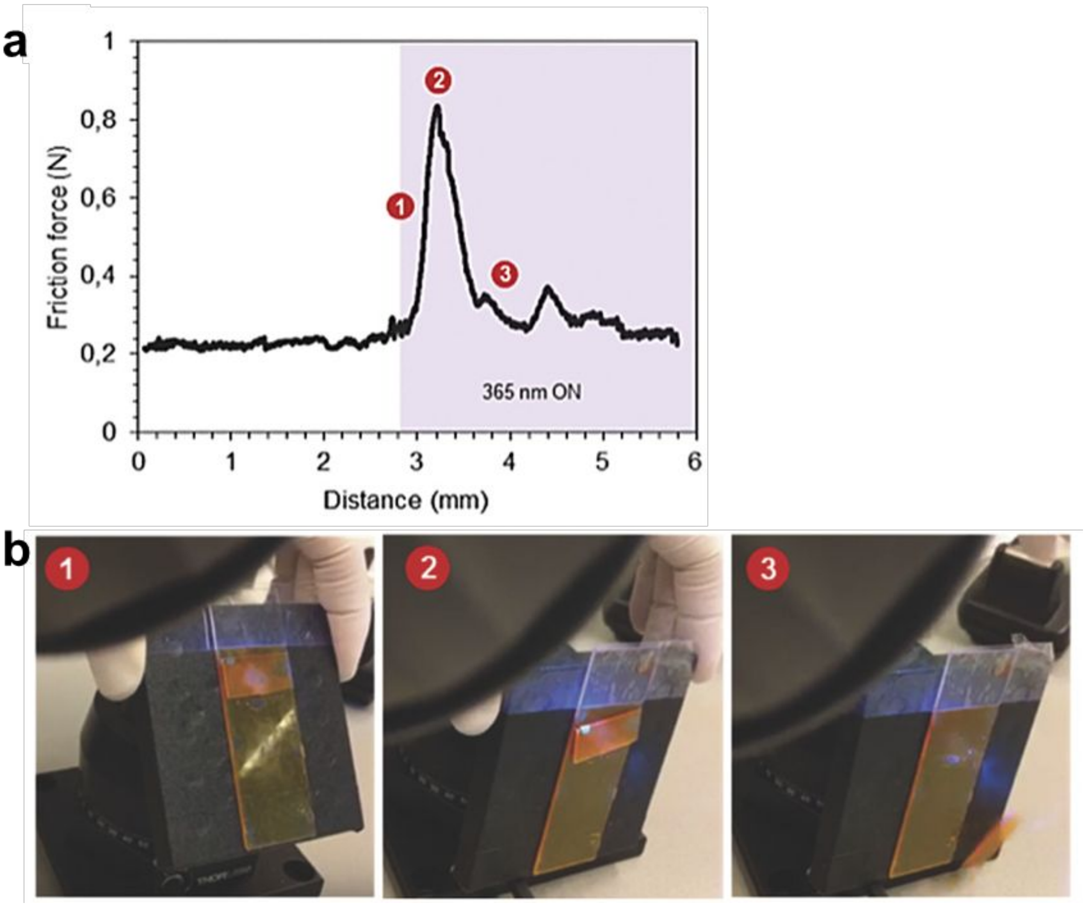

Figure S6. (a) Footage of light-controlled adhesion and release on a LCN photo-sponge by capillary bridging. Reproduced with permission from ref 22. Copyright 2018 John Wiley and Sons. (b) A graph

of the corresponding film friction in each snapshot. Reproduced with permission from ref 22. Copyright 2018 John Wiley and Sons.
